# Factors associated with reduced kidney function in hospitalized children with dehydration

**DOI:** 10.3389/fped.2026.1917450

**Published:** 2026-10-09

**Authors:** Dovilė Ruzgienė, Georgios Stathoglou, Inga Ivaškevičienė, Augustina Jankauskienė

**Affiliations:** Faculty of Medicine, Vilnius University, Vilnius, Lithuania

**Keywords:** children, dehydration, ibuprofen, paracetamol, reduced kidney function, symptom duration

## Abstract

**Introduction:**

Dehydration-related kidney impairment is often underestimated. The study evaluated factors associated with reduced estimated glomerular filtration rate (eGFR) at admission in dehydrated children requiring intravenous fluids.

**Methods:**

A prospective observational study involved 1–17-year-old children hospitalized with moderate or severe dehydration. Patients were stratified by admission eGFR: Group A (eGFR <90 mL/min/1.73 m^2^), Group B (eGFR ≥90 mL/min/1.73 m^2^). Demographic and clinical characteristics, ibuprofen and paracetamol exposure, chronic diseases, long-term medication use, birth characteristics, and laboratory parameters were compared.

**Results:**

Among 179 children, 90 were in Group A and 89 in Group B. Pre-admission ibuprofen use was more frequent in Group A (65.6% vs. 47.2%; unadjusted odds ratio [OR] 2.13, 95% confidence interval [CI] 1.17–3.89), as was paracetamol use (52.2% vs. 31.5%; unadjusted OR 2.38, 95% CI 1.29–4.38). Symptom duration before hospitalization was longer in Group A (median 48 vs. 24 h, *p* < 0.001) but showed poor discriminatory ability (area under the curve [AUC] = 0.655, *p* < 0.001). Numerically higher cumulative paracetamol exposure was observed in Group A, but the between-group difference was not statistically significant (*p* = 0.095), as well as the exploratory discriminatory analysis. (AUC = 0.616, 95% CI 0.487–0.745, *p* = 0.077). Severe dehydration was not statistically significantly associated with reduced admission eGFR (adjusted OR 1.96, 95% CI 0.97–3.98, *p* = 0.062). Respiratory illness was independently associated with reduced admission eGFR (adjusted OR 5.49, 95% CI 1.58–19.03, *p* = 0.007). Other clinical and laboratory parameters did not differ.

**Conclusion:**

Prolonged symptoms before hospitalization and respiratory illnesses as the primary cause of dehydration were associated with reduced admission eGFR. Age, medication exposure, and comorbidities were not independently associated with reduced admission eGFR after adjustment; birth-weight distributions and laboratory parameters did not differ significantly between groups.

## 1 Introduction

Acute kidney injury (AKI) is a common and clinically significant complication in pediatric patients and is associated with increased morbidity, prolonged hospitalization, and unfavorable short- and long-term outcomes (1, 2). Although AKI is traditionally associated with critically ill children, intensive care unit admissions, and postoperative settings, kidney dysfunction may also occur in children hospitalized with common acute illnesses (3–8). In pediatric patients, reduced kidney perfusion resulting from dehydration represents an important mechanism contributing to impaired kidney function and AKI development.

Dehydration in children most commonly occurs in the setting of acute gastroenteritis but may also accompany other acute febrile illnesses associated with reduced oral intake and increased fluid losses. In the presence of volume depletion, compensatory renal mechanisms may become insufficient, resulting in decreased glomerular filtration and reduced estimated glomerular filtration rate (eGFR). Studies of children hospitalized with acute gastroenteritis have identified longer symptom duration, greater dehydration severity, and exposure to nonsteroidal anti-inflammatory drugs (NSAIDs), particularly ibuprofen, as factors associated with AKI (9–12). However, these findings are derived mainly from gastrointestinal illness, and the relationship of dehydration severity and delayed presentation with reduced admission eGFR across a broader range of common acute illnesses remains incompletely characterized.

AKI has also been reported in non-gastrointestinal febrile illnesses such as community-acquired pneumonia (13), but the combined roles of dehydration and pre-admission medication exposure in these settings remain insufficiently characterized. Evidence regarding other antipyretics, particularly paracetamol, is also limited in dehydrated children and has largely been derived from different clinical settings (14). Although AKI is known to occur in noncritically ill hospitalized children, previous studies have generally examined broad inpatient populations or specific disease and medication contexts rather than the combined clinical factors associated with reduced admission eGFR in otherwise noncritically ill dehydrated children (8). Therefore, the combined contribution of dehydration severity, delayed presentation, NSAID and paracetamol exposure, and underlying illness category to reduced admission eGFR remains insufficiently described. Identifying these factors may improve recognition of reduced estimated kidney function at admission.

Therefore, this prospective observational study aimed to evaluate the factors associated with reduced admission eGFR in dehydrated pediatric patients requiring hospitalization and intravenous fluid therapy. Specific demographic, anamnestic, clinical, medication-related, and laboratory variables were assessed and compared between children with reduced and normal admission eGFR to identify factors associated with reduced admission eGFR in this clinically common but understudied pediatric population.

## 2 Materials and methods

This prospective observational study included children and adolescents aged 1–17 years who were admitted to the Pediatrics Center of Vilnius University Hospital Santaros Klinikos (VULSK) in Vilnius, Lithuania, from November 2024 to March 2026.

The study inclusion criteria were:

- Age 1–17 years;
- Moderate or severe dehydration requiring intravenous fluid therapy due to an acute illness;
- Signed informed consent.

Children having chronic kidney disease (CKD), oncohematological diseases, or diabetes mellitus were excluded from the study.

Participants were stratified into two groups based on admission eGFR: Group A comprised children with reduced admission eGFR (eGFR < 90 mL/min/1.73 m^2^), and Group B comprised children with normal admission eGFR (eGFR ≥ 90 mL/min/1.73 m^2^). The eGFR was calculated using the 2009 Chronic Kidney Disease in Children (CKiD) bedside Schwartz equation: eGFR (mL/min/1.73 m^2^) = 36.5 × height (cm)/serum creatinine (μmol/L) (15).

Serum creatinine was measured upon admission, before intravenous fluid therapy, in all 179 participants using an enzymatic assay traceable to isotope-dilution mass spectrometry (IDMS).

Previous renal-function data were available in 82 participants; in the remaining participants, CKD was excluded based on available medical records and clinical history, although previously unrecognized CKD could not be completely excluded. The severity of dehydration was classified in accordance with our institution's pediatric protocol for the management of fluid, electrolyte, and acid–base disturbances. This protocol defines the clinical criteria used to categorize the severity of dehydration; these criteria are detailed in Supplementary Table S1. The same criteria were applied uniformly to all children, irrespective of the underlying etiology of dehydration. According to the protocol, intravenous fluid therapy is indicated only for moderate or severe dehydration; therefore, only children with moderate or severe dehydration were eligible for inclusion. Children with mild dehydration were excluded.

Participants were categorized into three age groups: 1–6 years, 7–11 years, and 12–17 years. Based on birth weight, children were classified according to World Health Organization (WHO) standards as very low birth weight (<1,500 g), low (1,500–<2,500 g), normal birth weight (2,500–<4,000 g), or high birth weight (≥4,000 g). Only participants with recorded birth-weight data were included in the birth-weight analysis; missing values were not imputed.

Factors associated with reduced admission eGFR were assessed using anamnestic and laboratory data collected during hospitalization. Anamnestic variables included age, birth weight, gestational age, duration and characteristics of symptoms prior to hospitalization, pre-admission exposure to ibuprofen and paracetamol, presence of chronic diseases, and long-term medication use. Long-term medication use was defined as continuous use for at least three months. Chronic disease was operationally defined as an ongoing pre-existing condition recorded before the index hospitalization, separately from previous resolved illnesses and acute conditions documented during hospitalization. The duration of symptoms prior to hospitalization was recorded in hours and compared between age groups. Clinical symptoms were grouped into three categories: Group 1—diarrhea and vomiting without fever; Group 2—diarrhea, vomiting, and fever; and Group 3—fever without diarrhea or vomiting, associated with other febrile illnesses. For ibuprofen and paracetamol, pre-admission use, number of administrations, reported individual doses (mg), and cumulative dose (mg) were recorded. Cumulative exposure (mg/kg) was calculated by dividing the cumulative dose by admission weight. Concomitant exposure was defined as reported use of both medications. Anamnestic and medication-exposure data were obtained through interviews with the patients' parents or legal guardians by a single investigator.

The leading diagnoses in both groups were analyzed and categorized into three groups: gastrointestinal, respiratory infections, and other miscellaneous diagnoses. The “other” category comprised diagnoses not assigned to the first two categories; these diagnoses are listed individually in Supplementary Table S3. Laboratory variables included blood pH, serum bicarbonate, potassium, sodium, hemoglobin, and hematocrit levels. Other markers of kidney dysfunction, such as blood urea nitrogen and uric acid, were not measured. All laboratory tests were conducted upon admission, prior to the initiation of fluid therapy. The laboratory analyses were performed at the VULSK Laboratory Medicine Center using standardized methods.

Post-rehydration creatinine/eGFR measurements were not systematically available.

The study was approved by the Vilnius Regional Bioethics Committee, no. 2024/11-1614-1073. Written informed consent was obtained from parents or legal guardians and from children aged 12 years or older.

### 2.1 Statistical analysis

Study size was guided by comparable pediatric dehydration studies. Assuming 80% power, a two-sided *α* of 0.05, and equal group sizes, the estimated minimum sample was 118 participants (59 per group). Continuous variables were presented as mean ± standard deviation (SD), and categorical variables were presented as counts (percentages). Variables with markedly skewed distributions were summarized using the median and interquartile range (IQR) and compared using the two-sided Mann–Whitney U test. For continuous variables without marked skewness, group comparisons were performed using the independent-samples Student's t-test, or Welch's *t*-test when variances were unequal. Categorical variables were compared using the *χ*^2^ (chi-squared) test or Fisher's exact test. For the principal binary medication-exposure comparisons, unadjusted odds ratios (ORs), 95% confidence intervals (CIs), and absolute differences in the proportion with reduced admission eGFR were calculated. The age-category distribution was evaluated using an overall 3 × 2 Pearson chi-square test. Because the separated birth-weight categories contained sparse cells, their overall distribution was compared using the Fisher–Freeman–Halton exact test. The proportion of missing birth-weight data between groups was compared using a 2 × 2 Pearson chi-square test. Pearson and Spearman correlation analyses were used to assess the association between age and symptom duration prior to hospitalization. Symptom duration across age groups was compared using the Kruskal–Wallis test. Symptom duration was log₂-transformed because of its right-skewed distribution; therefore, its adjusted odds ratio represents the change associated with each doubling of symptom duration. Receiver operating characteristic (ROC) analyses were exploratory and were not prespecified. The area under the curve (AUC) and 95% confidence intervals (CIs) were reported. Because no internal or external validation was performed, discriminatory performance was considered apparent. A multivariable binary logistic regression model was fitted using reduced admission eGFR status (Group A versus Group B) as the dependent variable. The model included age, sex, symptom duration, illness category, pre-admission ibuprofen exposure, pre-admission paracetamol exposure, chronic disease, and dehydration severity (severe versus moderate). Gastrointestinal illness was used as the reference category. Adjusted odds ratios (ORs) with 95% confidence intervals (CIs) were reported, and apparent model discrimination was summarized using the area under the receiver operating characteristic curve. A two-sided *p*-value < 0.05 was considered statistically significant. Original descriptive and univariable analyses were performed using IBM SPSS Statistics for Macintosh, version 31.0 (IBM Corp., Armonk, NY, USA). Multivariable and sensitivity analyses and independent verification of the revised results were conducted using Python 3.12.14 (Python Software Foundation), with pandas 2.2.3 and NumPy 2.3.5.

## 3 Results

Totally 179 children were included: 90 with reduced admission eGFR (Group A) and 89 with normal admission eGFR (Group B). The detailed recruitment process is presented in the flowchart provided in the Supplementary Figure S1. The overall distribution of age groups did not differ significantly between Groups A and B [*χ*^2^(2) = 4.91, *p* = 0.086]. Similarly, baseline characteristics, including sex distribution, the prevalence of chronic diseases, and long-term medication use, did not differ significantly between the groups. Baseline characteristics of both groups are presented in Table 1.

**Table 1:** Baseline characteristics of group A and group B.

Variable	Group A (*n* = 90), *n* (%)	Group B (*n* = 89), *n* (%)	*p*-value
Sex			0.83
Male	49 (54.4%)	47 (52.8%)	
Female	41 (45.6%)	42 (47.2%)	
Age, years			
Mean ± SD	8.57 ± 4.48	8.09 ± 4.51	0.48
Median age, years (IQR)	8.5 (7)	7.0 (7)	—
Age groups			0.086
1–6 years	27 (30.0%)	41 (46.1%)	
7–11 years	32 (35.6%)	24 (27.0%)	
12–17 years	31 (34.4%)	24 (27.0%)	
Primary illness category			0.002
Gastrointestinal	51 (56.7%)	69 (77.5%)	
Respiratory	19 (21.1%)	4 (4.5%)	
Other	20 (22.2%)	16 (18.0%)	
Chronic diseases	32 (35.6%)	23 (25.8%)	0.16
Long-term medication use	11 (12.2%)	8 (9.0%)	0.48

On admission, median serum creatinine was 63.0 µmol/L (IQR 53.2–73.0) in Group A and 39.0 µmol/L (IQR 30.0–49.0) in Group B; the corresponding median eGFR values were 81.7 mL/min/1.73 m^2^ (IQR 75.3–86.0) and 124.7 mL/min/1.73 m^2^ (IQR 111.0–139.0), respectively. Admission eGFR values in Group A are presented in Figure 1.

**Figure 1:**
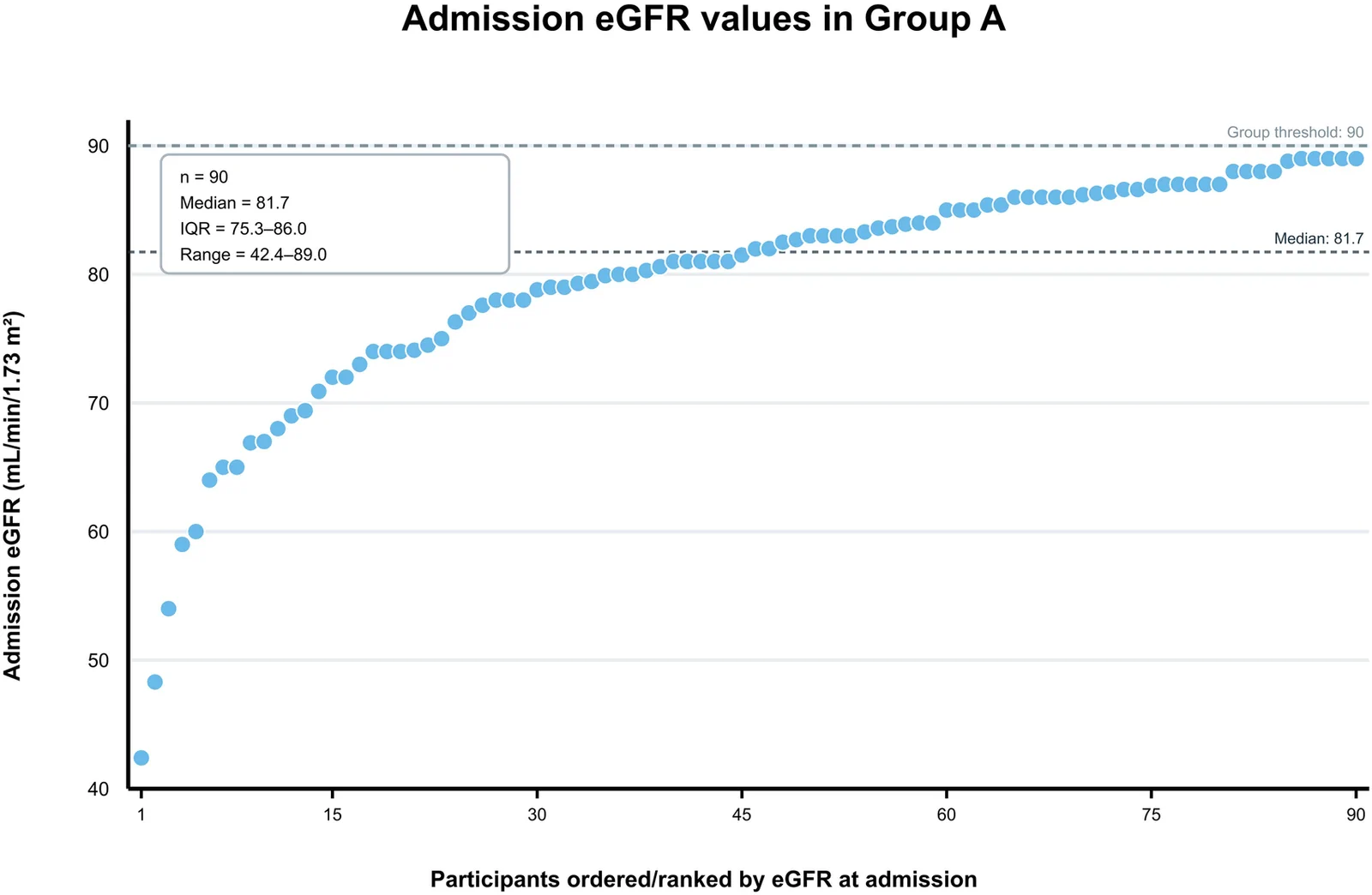
Admission eGFR values in group A.

In Group A, 58 of 90 children (64.4%) had moderate dehydration and 32 (35.6%) had severe dehydration. In Group B, 62 of 89 children (69.7%) had moderate dehydration and 27 (30.3%) had severe dehydration. The distribution of dehydration severity did not differ significantly between the groups [*χ*^2^(1) = 0.55, *p* = 0.458].

Gastrointestinal illnesses were the most common diagnosis, occurring in 51 of 90 children (56.7%) in Group A and 69 of 89 children (77.5%) in Group B. Respiratory illnesses were observed in 19 children (21.1%) in Group A and 4 children (4.5%) in Group B, whereas other illnesses were reported in 20 (22.2%) and 16 (18.0%) children, respectively. The distribution of illness categories differed significantly between the two groups [Pearson *χ*^2^(2) = 12.92, *p* = 0.002] (Table 1). The individual primary diagnoses included in the “other” category are listed in Supplementary Supplementary Table S3. Chronic diseases were recorded in 32/90 children (35.6%) in Group A and 23/89 children (25.8%) in Group B, with no significant difference between groups [Pearson *χ*^2^(1) = 1.98, *p* = 0.159]. The recorded conditions were heterogeneous and included allergic/atopic (most common in both groups), cardiovascular, neurological/developmental, urogenital, metabolic abnormalities, and other isolated medical conditions. As most individual conditions occurred in only one child, condition-specific statistical comparisons were not performed.

Birth-weight data were available for 77/90 children (85.6%) in Group A and 84/89 (94.4%) in Group B. Birth-weight data were unavailable for 13/90 children (14.4%) in Group A and 5/89 (5.6%) in Group B. The proportion of unavailable birth-weight data was higher in Group A than in Group B [Pearson *χ*^2^(1) = 3.85, *p* = 0.0496]. Among children with available data, the distribution of birth-weight categories did not differ significantly between groups (Fisher–Freeman–Halton exact *p* = 0.883). Most children in both groups were born at term. A more detailed description is provided in Table 2.

**Table 2:** Distribution of gestational age and birth weight in group A and group B.

Variable	Category	Group A (n), *n* (%)	Group B (n), *n* (%)	*p*-value
Gestational age	Term	85/90 (94.4%)	85/89 (95.5%)	1.00
	Preterm	5/90 (5.6%)	4/89 (4.5%)	
Birth weight	Very low (<1,500 g)	2/77 (2.6%)	1/84 (1.2%)	0.883
	Low (1,500 ≤ 2,500 g)	4/77 (5.2%)	5/84 (6.0%)	
	Normal (2,500–<4,000 g)	59/77 (76.6%)	67/84 (79.8%)	
	High (≥4,000 g)	12/77 (15.6%)	11/84 (13.1%)	

Pre-admission ibuprofen use was more frequent in Group A than in Group B (65.6% vs. 47.2%; unadjusted OR 2.13, 95% CI 1.17–3.89; *p* = 0.013), as was paracetamol use (52.2% vs. 31.5%; unadjusted OR 2.38, 95% CI 1.29–4.38; *p* = 0.005). The absolute differences in the proportion of children with reduced admission eGFR between exposed and unexposed children were 18.7 percentage points (95% CI 4.2–33.2) for ibuprofen and 21.3 percentage points (95% CI 6.8–35.8) for paracetamol. The median number of ibuprofen administrations was 3 (IQR 2–6) in Group A (*n* = 59) and 3 (IQR 2–5) in Group B (*n* = 42). For paracetamol, the corresponding values were 3 (IQR 1–6; *n* = 47) and 1.5 (IQR 1–3; *n* = 28), respectively. Median cumulative ibuprofen exposure was similar between groups: 23.2 (IQR 13.8–47.9) mg/kg in Group A and 23.4 (IQR 14.1–41.3) mg/kg in Group B. Median cumulative paracetamol exposure was 28.9 (IQR 15.3–60.2) mg/kg and 24.4 (IQR 13.0–34.8) mg/kg, respectively. Patterns of single- versus combined-drug use did not differ significantly between groups (*p* = 0.410). Cumulative paracetamol exposure was numerically higher in Group A, but the between-group difference was not statistically significant (Mann–Whitney *p* = 0.095). Its discriminatory performance was poor and did not reach statistical significance (AUC = 0.616, 95% CI 0.487–0.745; *p* = 0.077). Cumulative ibuprofen exposure also did not differ between the groups (Mann–Whitney *p* = 0.842) and showed no discriminatory ability (AUC = 0.512, 95% CI 0.397–0.627; *p* = 0.839). As these ROC analyses were exploratory and unvalidated, no medication-dose threshold was interpreted as clinically meaningful. None of the patients exceeded recommended age/weight-based doses of ibuprofen or paracetamol. The distributions of cumulative paracetamol and ibuprofen doses per kilogram are shown in Figure 2. Detailed pre-admission medication-exposure characteristics are presented in Table 3.

**Figure 2:**
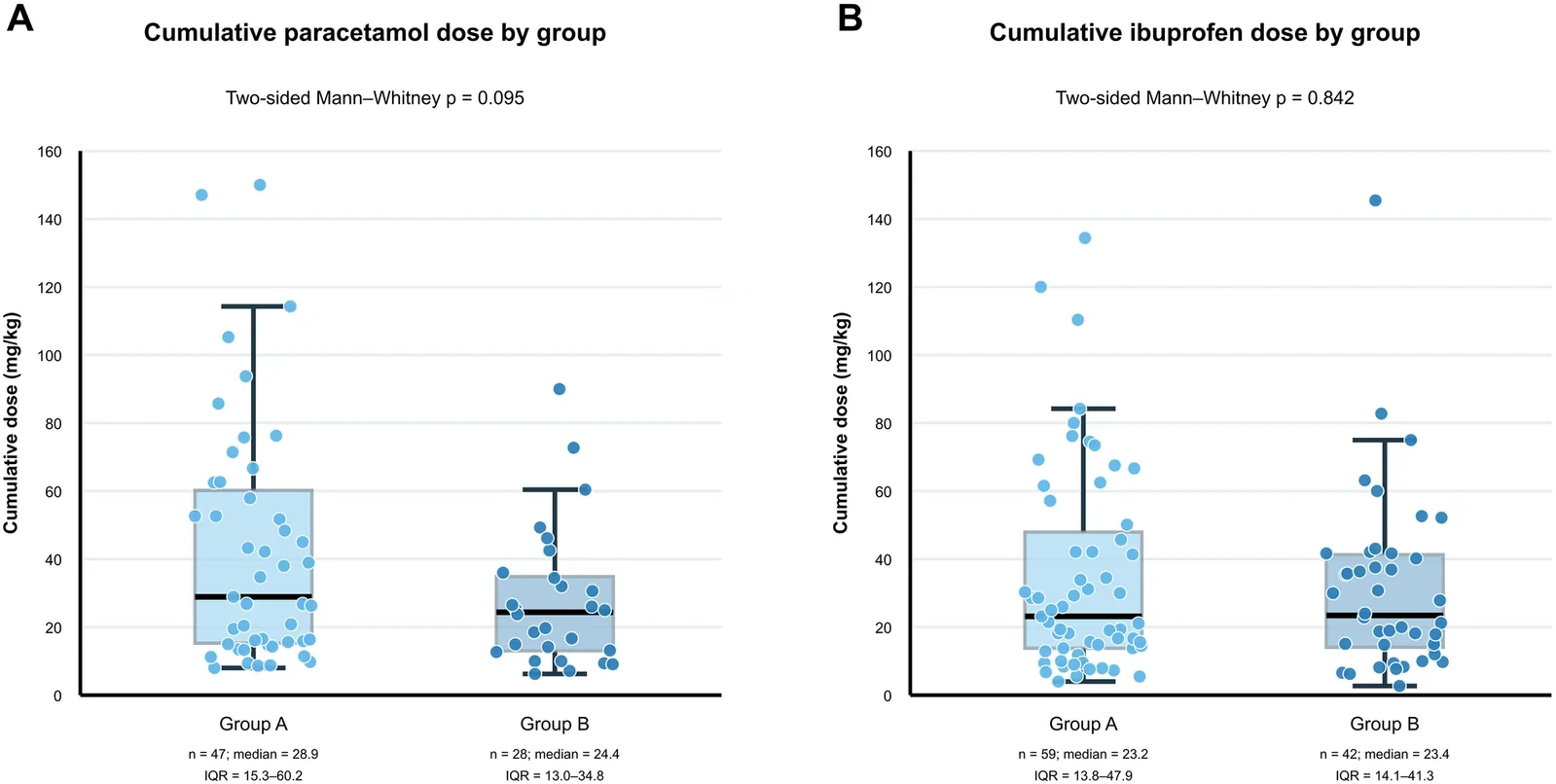
Cumulative paracetamol and ibuprofen doses in group A and group B.

**Table 3:** Pre-admission medication exposure characteristics in groups A and *B.*

Variable	Group A	Group B	*p*-value
Ibuprofen			
Number of administrations, median (IQR)	3 (2–6), *n* = 59	3 (2–5), *n* = 42	0.452
Cumulative exposure, mg/kg, median (IQR)	23.2 (13.8–47.9), *n* = 59	23.4 (14.1–41.3), *n* = 42	0.842
Paracetamol			
Number of administrations, median (IQR)	3 (1–6), *n* = 47	1.5 (1–3), *n* = 28	0.023
Cumulative exposure, mg/kg, median (IQR)	28.9 (15.3–60.2), *n* = 47	24.4 (13.0–34.8), *n* = 28	0.095
Medication-use pattern^a^			0.410
Both medications	36/70 (51.4%)	20/50 (40.0%)	—
Ibuprofen only	23/70 (32.9%)	22/50 (44.0%)	__
Paracetamol only	11/70 (15.7%)	8/50 (16.0%)	__

a Medication-use pattern percentages were calculated among participants who received at least one of the two medications (Group A, *n* = 70; Group B, *n* = 50).

The duration of symptoms prior to hospitalization was longer in Group A (median 48 [IQR 24–72] vs. 24 [IQR 11–48] hours, *p* < 0.001) but demonstrated poor discriminatory ability in an exploratory ROC analysis (AUC = 0.655, 95% CI 0.575–0.734, *p* < 0.001) (Figure 3).

**Figure 3:**
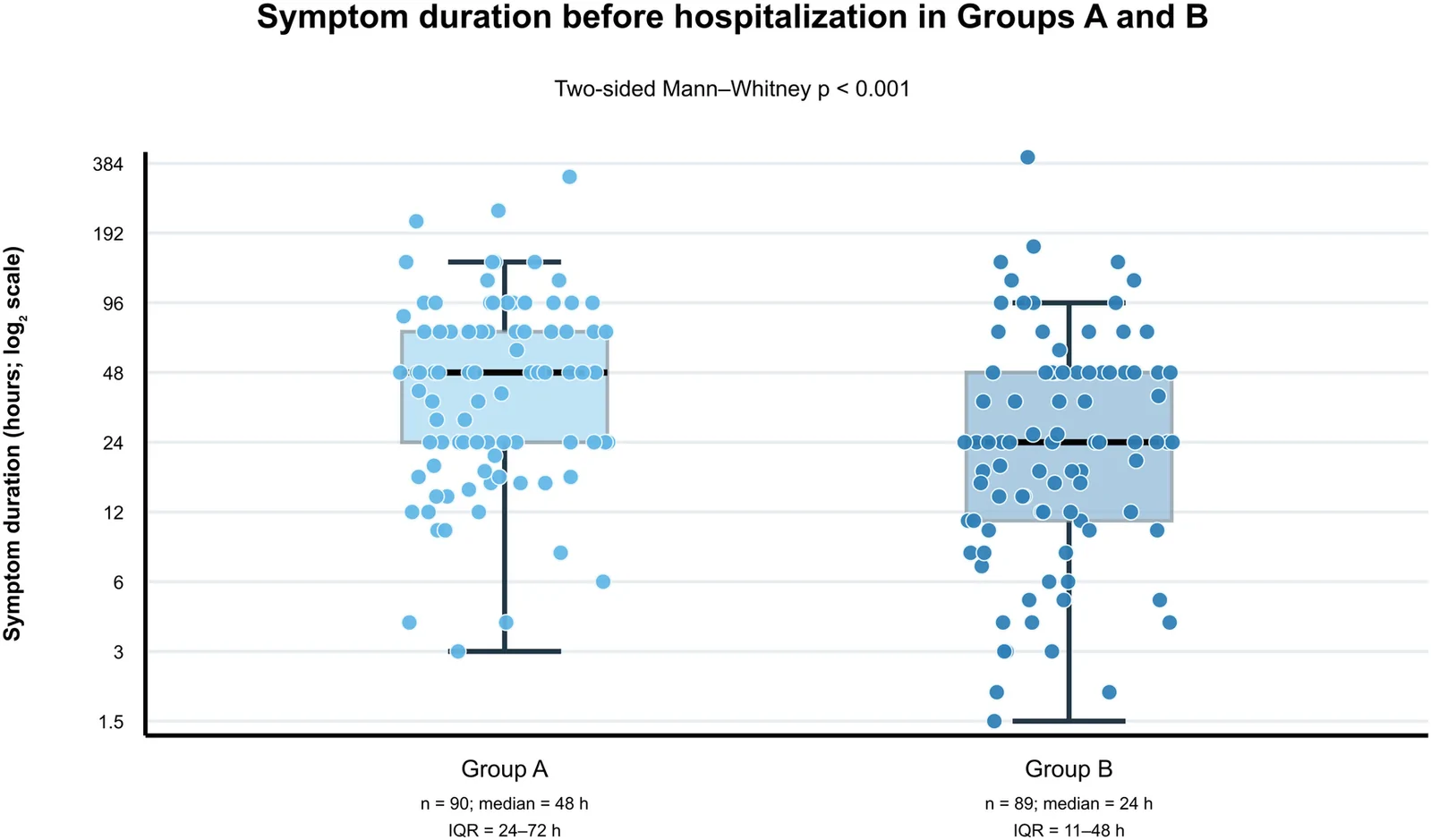
Symptom duration prior to hospitalization in group A and group B.

The multivariable binary logistic regression model for reduced admission eGFR was statistically significant [likelihood-ratio *χ*^2^(9) = 32.58, *p* < 0.001] and demonstrated acceptable apparent discrimination (AUC = 0.725). Each doubling of symptom duration was associated with higher odds of reduced admission eGFR (adjusted OR 1.46, 95% CI 1.14–1.87, *p* = 0.003). Compared with gastrointestinal illness, respiratory illness was independently associated with higher odds of reduced admission eGFR (adjusted OR 5.49, 95% CI 1.58–19.03, *p* = 0.007). Severe dehydration was not statistically significantly associated with reduced admission eGFR (adjusted OR 1.96, 95% CI 0.97–3.98, *p* = 0.062). Neither pre-admission ibuprofen exposure (adjusted OR 0.97, 95% CI 0.47–2.03, *p* = 0.939) nor paracetamol exposure (adjusted OR 1.62, 95% CI 0.80–3.28, *p* = 0.183) was significantly associated with reduced admission eGFR after adjustment. Similarly, age, sex, other illness categories, and chronic disease were not significantly associated with reduced admission eGFR in the adjusted model. All 179 participants had complete data for the variables included in the multivariable model. Complete multivariable regression results are presented in Table 4.

**Table 4:** Multivariable logistic regression analysis of factors associated with reduced admission eGFR.

Predictor	Adjusted OR	95% CI	*p*-value
Age, per 1-year increase	1.02	0.95–1.10	0.559
Sex, male vs. female	1.28	0.66–2.47	0.463
Symptom duration, per doubling	1.46	1.14–1.87	0.003
Respiratory vs. gastrointestinal illness	5.49	1.58–19.03	0.007
Other vs. gastrointestinal illness	1.29	0.56–2.96	0.549
Ibuprofen exposure, yes vs. no	0.97	0.47–2.03	0.939
Paracetamol exposure, yes vs. no	1.62	0.80–3.28	0.183
Chronic disease, present vs. absent	1.70	0.83–3.46	0.145
Severe vs. moderate dehydration	1.96	0.97–3.98	0.062

Model *n* = 179. Likelihood-ratio *χ*^2^(9) = 32.58, *p* < 0.001; apparent AUC = 0.725.

No correlation was observed between age and symptom duration before hospitalization (Spearman's rho = 0.040, *p* = 0.59; Pearson's r = 0.060, *p* = 0.42). Symptom duration did not differ significantly across age categories [Kruskal–Wallis H(2) = 1.60, *p* = 0.449], with median durations of 25 h (IQR 14–48) in children aged 1–6 years, 36 h (IQR 14.75–51) in those aged 7–11 years, and 36 h (IQR 17–96) in those aged 12–17 years.

Symptom patterns were similarly distributed between groups and were not statistically significantly associated with reduced admission eGFR (Table 5).

**Table 5:** Distribution of symptom categories in group A and group B.

Variable	Symptom category	Group A (*n* = 90), *n* (%)	Group B (*n* = 89), *n* (%)	*p*-value
Symptom pattern	Diarrhea + vomiting without fever	12 (13.3%)	14 (15.7%)	0.71
	Diarrhea + vomiting with fever	28 (31.1%)	23 (25.8%)	
	Fever without diarrhea/vomiting	50 (55.6%)	52 (58.4%)	

Laboratory parameters, including blood pH, sodium, potassium, bicarbonate, hemoglobin, and hematocrit, were similar between groups (all *p* > 0.05). Available-case denominators were 176 for blood pH and bicarbonate, 175 for potassium and sodium, and 177 for hemoglobin and hematocrit.

## 4 Discussion

Most studies investigating pediatric AKI primarily focus on critically ill children, patients admitted to intensive care units, or children undergoing cardiac surgery, where AKI is commonly associated with hemodynamic instability, multiple organ dysfunction, and exposure to intensive therapies (3–7). However, one of the most common etiologies of AKI is kidney hypoperfusion resulting from dehydration (16). In the pediatric population, dehydration is most frequently caused by acute gastroenteritis, which, through mechanisms of hypoperfusion, accounts for approximately 12% of all cases of AKI in children (17). Our study specifically focused on dehydrated children requiring intravenous fluid therapy, representing a clinically common but frequently underestimated population in whom reduced admission eGFR may occur (1).

Previous studies have demonstrated that exposure to NSAIDs, particularly ibuprofen, is a biologically plausible and clinically recognized risk factor for AKI in dehydrated children, including when administered at standard therapeutic doses (9–12, 18, 19). In our cohort, pre-admission ibuprofen and paracetamol use was more frequent among children with reduced admission eGFR in unadjusted comparisons. However, these medications should not be interpreted equivalently. The association between ibuprofen and AKI during volume depletion is supported by existing pediatric evidence, whereas the finding for paracetamol in this study was exploratory (9–12). Higher cumulative paracetamol exposure was observed in unadjusted analyses; however, its discriminatory performance was poor and did not reach statistical significance. This finding may reflect confounding by indication because children with fever or longer illness duration may be more likely to receive paracetamol. Therefore, the paracetamol findings do not establish an independent or causal relationship (10, 12, 20, 21).

The overall age-group distribution did not differ significantly between groups, but the numerically higher proportions of children aged 7–11 and 12–17 years were in Group A. Although the exact explanation for this observation remains unclear, one possible hypothesis is that parents recognize dehydration symptoms in younger children earlier, leading to more timely medical evaluation and treatment. Associations between delayed presentation, prolonged symptom duration, and AKI development have been reported in previous pediatric studies investigating dehydration-related kidney injury (9, 10, 13). However, no correlation was observed between age and symptom duration in our study. Another important observation from our study was that febrile acute respiratory illnesses were the second most common diagnosis among hospitalized children, after gastrointestinal infections. While dehydration associated with gastroenteritis is usually clinically anticipated, fluid loss related to fever and reduced oral intake during respiratory infections may be underestimated both by parents and healthcare providers. One possible explanation is that persistent fever and higher respiratory rate may contribute to greater insensible fluid losses, particularly in pediatric patients, potentially contributing to clinically significant dehydration and reduced admission eGFR even in the absence of significant gastrointestinal symptoms (9). However, this mechanism remains hypothetical and cannot be established from our study data. Nevertheless, this finding suggests that clinicians should maintain careful assessment of hydration status not only in children presenting with vomiting and diarrhea, but also in febrile respiratory illnesses, especially when nephrotoxic medications such as ibuprofen are administered (19).

Previous studies have demonstrated that comorbid conditions and long-term medication exposure, particularly to potentially nephrotoxic drugs, are important contributors to pediatric AKI development, especially in hospitalized and critically ill children (3, 4, 12, 14, 22). In our study, chronic diseases and long-term medication use were numerically more frequent in Group A than in Group B, but neither difference was statistically significant. This may indicate that other factors and delayed presentation have a greater influence on reduced estimated kidney function at admission than the presence of chronic medical conditions in otherwise noncritically ill pediatric patients.

Research on children hospitalized with acute gastroenteritis identified lower serum bicarbonate levels as a risk factor associated with AKI (9); however, routine laboratory parameters did not differ significantly between groups in our cohort. This finding may suggest that reduced admission eGFR can occur without clear abnormalities in the routine laboratory parameters assessed, or that clinical factors such as dehydration severity and timing of presentation may play a greater role (9).

In our study, severe dehydration was not statistically significantly associated with reduced admission eGFR after adjustment. This finding differs from previous pediatric studies, in which greater dehydration severity was associated with AKI. Dehydration >5% was reported as an independent predictor of AKI in children hospitalized with acute gastroenteritis, while severe dehydration was also independently associated with AKI in infants with diarrhea (9, 23). These differences may reflect variation in study populations and outcome definitions, as our cohort included children with dehydration due to various acute illnesses and evaluated reduced admission eGFR rather than AKI. In addition, because all children in our study had at least moderate dehydration requiring intravenous fluid therapy, the restricted range of dehydration severity may have reduced our ability to detect an independent association.

Earlier investigations have suggested that AKI may be associated with prematurity and low birth weight (24), which we also aimed to evaluate in our study. However, our cohort was predominantly composed of children born at term and with normal birth weight. Therefore, we cannot confirm or exclude an association between these perinatal factors and reduced admission eGFR. In our data, no significant differences were observed between the groups.

Our study has several important strengths. First, the study is prospective and specifically focused on dehydrated pediatric patients with common acute pediatric illnesses, a clinically common but frequently underrecognized population in whom reduced admission eGFR may occur. Additionally, all clinical and anamnestic data were collected by a single investigator, ensuring a structured and consistent approach to data acquisition throughout the study period.

Nevertheless, several limitations should be acknowledged. The relatively small sample size may have limited the statistical power to detect associations in some analyses. The adjusted estimate for respiratory illness was based on relatively few participants (19 in Group A and 4 in Group B) and had a wide confidence interval, indicating imprecision. The birth-weight comparison may be biased by greater missingness in Group A than in Group B. Furthermore, the study was conducted in a single tertiary-care center and included only patients with moderate and severe dehydration. Consequently, the findings may not be generalizable to children with mild dehydration managed with oral rehydration, those treated in primary care settings, patients presenting to emergency departments who are discharged without admission, or children managed within different healthcare systems. Creatinine-based eGFR should be interpreted cautiously during acute dehydration, as serum creatinine may not be at steady state. Accordingly, a reduced admission eGFR may reflect transient hemodynamic changes rather than underlying structural kidney injury and should not be interpreted as evidence of intrinsic kidney damage. Baseline pre-illness creatinine values were not available for all patients, limiting assessment of changes from baseline. Because post-rehydration creatinine/eGFR measurements were not systematically available, the study could not assess the reversibility of reduced admission eGFR across the cohort. Finally, information regarding medical history, symptom duration, and pre-admission medication exposure was obtained through interviews with parents or legal guardians. Although many respondents referred to written records documenting medication doses and frequency of administration, the possibility of recall bias cannot be entirely excluded.

## 5 Conclusions

Reduced admission eGFR in dehydrated pediatric patients is a clinically important but potentially underestimated condition, particularly in the setting of prolonged symptoms and exposure to NSAIDs such as ibuprofen during volume depletion. Although pre-admission ibuprofen and paracetamol use was more frequent in the reduced admission eGFR group in unadjusted comparisons, neither medication remained independently associated with reduced admission eGFR after adjustment. Longer duration of symptoms before hospitalization was associated with reduced admission eGFR but demonstrated poor discriminatory ability. While gastrointestinal infections remained the predominant diagnosis, febrile respiratory illnesses also represented an important clinical context associated with dehydration and reduced admission eGFR. Severe versus moderate dehydration was not statistically significantly associated with reduced admission eGFR in the adjusted analysis. Early recognition of dehydration and consideration of medication exposure are clinically relevant when assessing children hospitalized with acute illnesses. Further, larger-scale multicenter prospective studies with multivariable adjustment are needed to clarify factors independently associated with reduced admission eGFR in dehydrated children.

## Data Availability

The original contributions presented in the study are included in the article/supplementary material, further inquiries can be directed to the corresponding author.
