# Supplementary material for: Factors associated with reduced kidney function in hospitalized children with dehydration
Source: Front Pediatr. 2026 Oct 9;14:1917450. doi: 10.3389/fped.2026.1917450 (PMC13690956; doi:10.3389/fped.2026.1917450)
Supplement: Supplementary file 2 [file Image2.pdf]

Supplementary Figure S2. Participant recruitment and study-group allocation

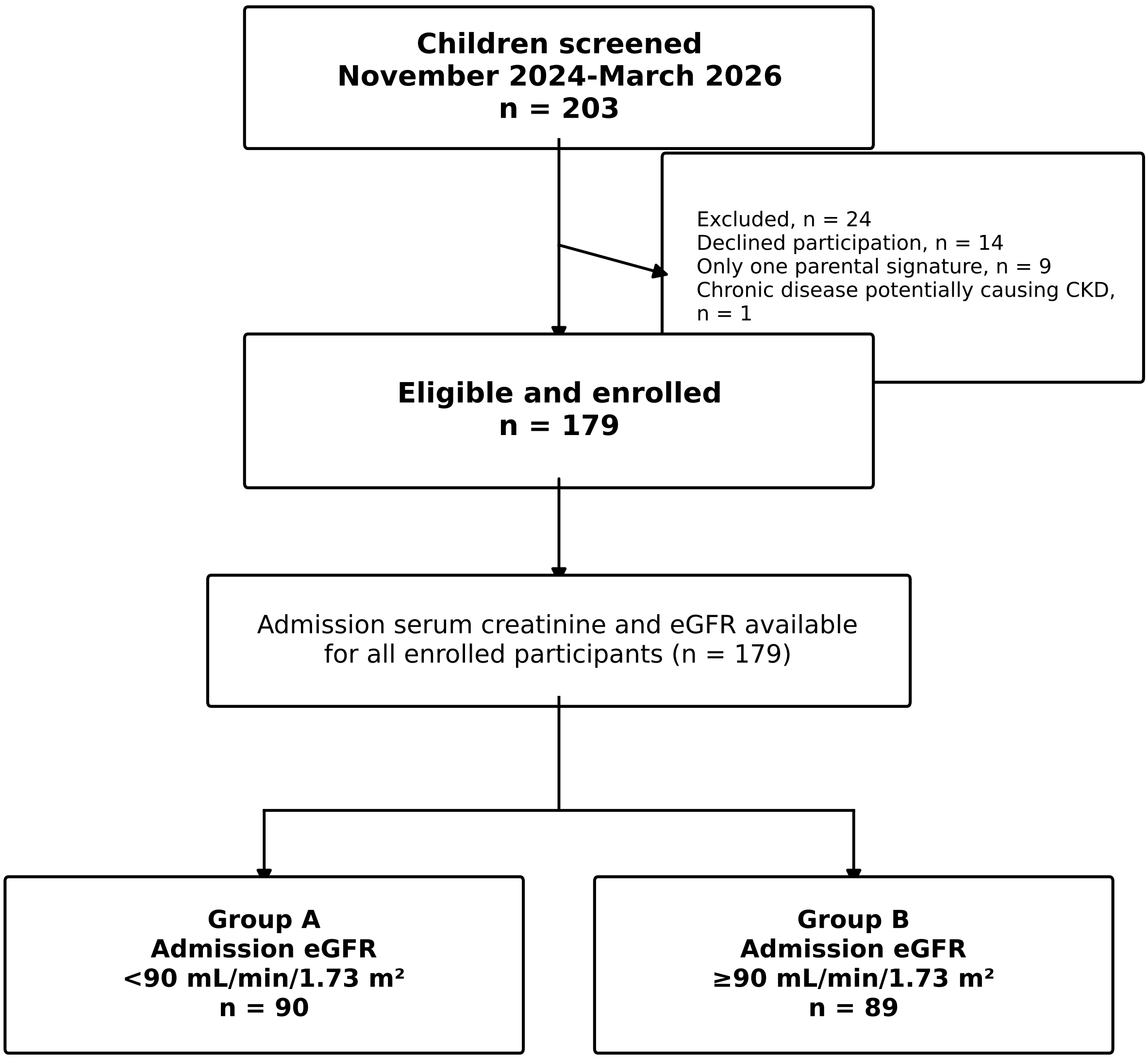

Availability of secondary variables varied; corresponding denominators are reported where applicable.

CKD, chronic kidney disease; eGFR, estimated glomerular filtration rate.
