# Supplementary material for: Factors associated with reduced kidney function in hospitalized children with dehydration
Source: Front Pediatr. 2026 Oct 9;14:1917450. doi: 10.3389/fped.2026.1917450 (PMC13690956; doi:10.3389/fped.2026.1917450)
Supplement: Supplementary file 3 [file Table1.docx]

Supplementary Material

# Supplementary Table S1. The evaluation of dehydration severity used in the study.

| **Symptom** | **Moderate dehydration (6-9% loss of body weight)** | **Severe dehydration (≥10% loss of body weight)** |
| --- | --- | --- |
| Mental status | Fatigued or restless, irritable | Apathetic, lethargic, unconscious |
| Heart rate | Increased | Tachycardia, with bradycardia in most severe cases |
| Quality of pulses | Decreased | Weak, thready, or impalpable |
| Systolic pressure | Normal to low | Low |
| Capillary refill | Prolonged | Prolonged, minimal |
| Mouth and tongue | Dry | Parched |
| Eyes | Slightly sunken | Deeply sunken |
| Tears | Decreased | Absent |
| Urine output | Decreased | Minimal |
