# Supplementary material for: Factors associated with reduced kidney function in hospitalized children with dehydration
Source: Front Pediatr. 2026 Oct 9;14:1917450. doi: 10.3389/fped.2026.1917450 (PMC13690956; doi:10.3389/fped.2026.1917450)
Supplement: Supplementary file 4 [file Table2.docx]

Supplementary Material

# Supplementary Table S3. Individual primary diagnoses included in the ‘other’ illness category.

| **Primary diagnosis** | **Group A (n = 90), n** | **Group B (n = 89), n** |
| --- | --- | --- |
| Acute appendicitis with generalized peritonitis | 1 | 0 |
| Acute hematogenous osteomyelitis | 0 | 1 |
| Acute myocarditis | 0 | 1 |
| Acute pyelonephritis | 3 | 0 |
| Acute tonsillitis | 1 | 1 |
| Epilepsy | 0 | 1 |
| Hepatitis A | 0 | 1 |
| Infectious myositis | 2 | 0 |
| Meningococcal infection | 1 | 0 |
| Pyoderma | 1 | 0 |
| Renal and perinephric abscess | 1 | 0 |
| Scarlet fever | 0 | 2 |
| Sepsis, unspecified | 1 | 0 |
| Sepsis due to group A Streptococcus | 0 | 1 |
| Syncope and collapse | 0 | 1 |
| Tick-borne encephalitis | 0 | 1 |
| Tuberculosis | 1 | 0 |
| Unspecified bacterial infection | 4 | 3 |
| Unspecified viral infection | 1 | 2 |
| Urinary tract infection, site not specified | 1 | 0 |
| Varicella | 2 | 1 |
| **Total** | **20** | **16** |

Note: Counts were obtained from the primary-diagnosis field. Chronic diseases were recorded separately. No diagnosis-specific inferential comparisons were performed because of sparse counts.
